# Supplementary material for: COVID-19-Related Neuropathic Pain: A Systematic Review and Meta-Analysis
Source: J Clin Med. 2023 Feb 20;12(4):1672. doi: 10.3390/jcm12041672 (PMC9966617; doi:10.3390/jcm12041672)
Supplement: Supplementary file 1 [file jcm-12-01672-s001.zip › Supplementary Table S2.pdf]

Study (NP)	Patient Characteristics	No. of included patients	NP assessment / Definition	Time since COVID-19 to NP development	NP related clinical risk factors
Miller et al, 2021 [25]	COVID-19 critical care patients (severe)<br>54.5 years (mean age)<br>80% hypertension, 46% type 2 diabetes, 53% obesity<br>32.5 days average critical care admission stay	256	Assessment via nerve conduction studies in hospital / clinical signs in neurology appointments	Not documented	Secondary neuropathic pain due to long ITU prone positioning
Ojeda et al, 2022 [28]	Hospitalised<br>55-76 years of age<br>Pain impacting daily living of patients<br>5 patients reported widespread body pain<br>Neuropathic pain reported mostly extremities and upper limb	65	Assessment via brief pain inventory and DN4 questionnaires	1 month after discharge	Pain intensity risk factor of chronic pain
Magdy et al, 2022 [31]	Steroids, azithromycin, other medications during COVID-19, different combinations for each patient<br>27 – 60 years<br>14 / 90 smokers	90	Assessment via neurological examination and DN4 questionnaire	Immediately following COVID-19 infection (upon hospitalisation) and delayed (3 months+)	Length of COVID-19 disease (results suggestive longer duration heightens NP risk)<br>Comorbidity, smoking not suggestive risk factors<br>Azithromycin use is a risk factor for neuropathic pain following COVID-19<br>Higher NFL levels<br>Depression presence<br>COVID-19 severity
Ocak & Sahin, 2022 [27]	Patients from COVID-19 outpatient clinic<br>232 males<br>50% +ve PCR<br>166 active smokers<br>36 regular alcohol users<br>Mean age younger for those reporting pain, vs those without pain<br>Comorbidities excluded<br>Different education backgrounds	440	Assessment via neuropathic pain questionnaire online	Not documented	Women higher pain intensity average<br>No difference between genders for neuropathic pain presence
Ali et al, 2022 [23]	Non-hospitalised<br>Long Covid	50	Assessment via phone or email questionnaire	14.8 months (average)	Autonomic dysfunction suggested as potential risk factor

	73% female<br>77% vaccinated for COVID-19<br>42.8 years mean age				
Oguz-Akarsu et al, 2021 [32]	41.8 years mean age (18-87 years)<br>Comorbidities (32.4%), e.g., hypertension, endocrine, diabetes, respiratory etc.<br>116 males (52.3%)<br>36 smokers (16.2%)<br>9 Immunocompromised (4.1%)<br>Widespread pain (36.4%)<br>Extremity pain (21.8%)	222	Assessment via interview, 1.5 – 3 months post COVID-19 infection (+ve PCR), by two experienced neurologists	0 – 3 months following COVID-19 infection	Strong association between neuropathic pain and headache / pain syndrome presence<br>Pain generally more frequent in females
Novak et al, 2022 [26]	Long-Covid<br>Autonomic, multisystemic symptoms	9	Assessment via skin biopsies, inflammatory and autoimmune markers, sudomotor testing,	4 – 6 weeks after initial COVID-19 infection	Long-Covid associations – SFN, respiratory dysregulation, chronic inflammation, cerebrovascular dysregulation
Jena et al, 2022 [24]	102 mild, 62 moderate, 18 severe COVID-19 infection<br>72.53% male (132 / 182)<br>32.6% diabetes (66 / 182)<br>Smoking and regular alcohol = 15 / 182<br>55 / 182 history of addiction<br>48.86 years mean age (13.98 years SD)	182	Assessment via DN4 questionnaire (collected data off the ward also)	Not documented	Higher COVID-19 severity (cytokine storm)<br>Higher BMI<br>History of Addiction
Shouman et al, 2021 [30]	All patients referred for autonomic testing following COVID-19 infection<br>41% male (11 / 27)	27	Assessment via sudomotor function, cardiovascular function, autonomic reflex screen, thermoregulatory sweat test	0 – 122 days following initial COVID-19 infection	Long-Covid overlap with ANS dysfunction and SFN<br>Neuropathic pain exacerbation and new-onset following COVID-19<br>Pre-existing neurological disease worsened by COVID-19 infection
Scherlinger et al, 2021 [29]	40% male (12 / 30)<br>10% diabetic (3 / 30)<br>Persistent symptoms had a cyclic pattern in 28 patients (93.3%)<br>40 years mean age (35 -54 years)	30	Assessment via physical examination (5 physicians), DN4 questionnaire, visual analogue scales, blood tests	Clinic evaluation 102 – 164 days following COVID-19 initial infection	COVID-19 infection increases anxiety / depression risk

			Also, fibromyalgia screen and rare systemic / autonomic disease screening	0-21 days symptom onset following initial COVID-19 infection	
Starace et al, 2021 [33]	69.7% of patients with TE and TR received treatments<br>Comorbidities not recorded<br>24 men, 104 women	128	Assessment via clinical observation in hair specialist clinic appointments	0 – 31 days symptom onset following initial COVID-19 infection (62.5%) (early onset)<br><br>12 weeks+ symptom onset following COVID-19 infection (47.8%) (late onset)	COVID-19 severity<br>Drug treatments potential risk factors (anticoagulants, hydroxychloroquine, azithromycin)
